# Rare Case of Iatrogenic Myocardial Infarction Induced by Use of Pyridostigmine

**DOI:** 10.7759/cureus.9849

**Published:** 2020-08-18

**Authors:** Muhammad Niazi, Qasim Z Iqbal, Zeeshan Zia, Saud Bin Abdul Sattar, James Lafferty

**Affiliations:** 1 Internal Medicine, Northwell Health-Staten Island University Hospital, New York, USA; 2 Cardiology, Northwell Health-Staten Island University Hospital, New York, USA

**Keywords:** iatrogenic myocardial infarction, pyridostigmine side effects, myasthenia gravis, paraneoplastic syndromes

## Abstract

Myasthenia gravis is an auto-immune disease that results in muscle weakness caused by antibodies released against acetylcholine receptors at the presynaptic membrane. Treatment options include acetylcholinesterase medications that cause a wide range of side-effects by increasing the concentration of acetylcholine at the synaptic cleft. One peculiar side effect seen is the precipitation of myocardial infarction caused by an excess of acetylcholine especially among elderly females. We present an interesting case of an 88-year-old female with a history of lung cancer newly diagnosed with paraneoplastic myasthenia gravis, started on treatment with prednisone 40 mg daily, and pyridostigmine 60 mg every six hours. She initially showed remarkable improvement in symptoms within a few hours, however, one day later, the patient developed sudden onset of chest pain radiating towards her left arm. A 12-lead electrocardiogram (EKG) showed diffuse ST-elevation in anterior leads and cardiac enzymes were found to be elevated. Pyridostigmine was stopped and the patient was started on heparin. The patient underwent cardiac catheterization which showed 50% stenosis in the right coronary artery (RCA) and 70% in the left anterior descending artery (LAD). The patient was monitored in the cardiac care unit (CCU) for 24 hours and later on discharged home on oral prednisone. It is a common practice to start treatment with anti-cholinesterase medications in newly diagnosed patients of myasthenia gravis, however, these medications can precipitate myocardial ischemia by coronary vasogenic spasm or by their arrhythmogenic effect. It is important to be aware of these outcomes while starting patients on these medications.

## Introduction

Myasthenia gravis is an autoimmune disorder that causes muscle weakness. It occurs due to antibodies released against acetylcholine receptors at the presynaptic membrane [1]. This common autoimmune disorder is treated by acetylcholinesterase inhibitors which work by increasing the acetylcholine at the synaptic cleft. This group of medication comes with several side effects, amongst which one of the very rare side effects is the precipitation of myocardial infarction caused by an excess of acetylcholine within the synaptic cleft. This is observed in the elderly population, primarily in females.

## Case presentation

**Figure 1:**
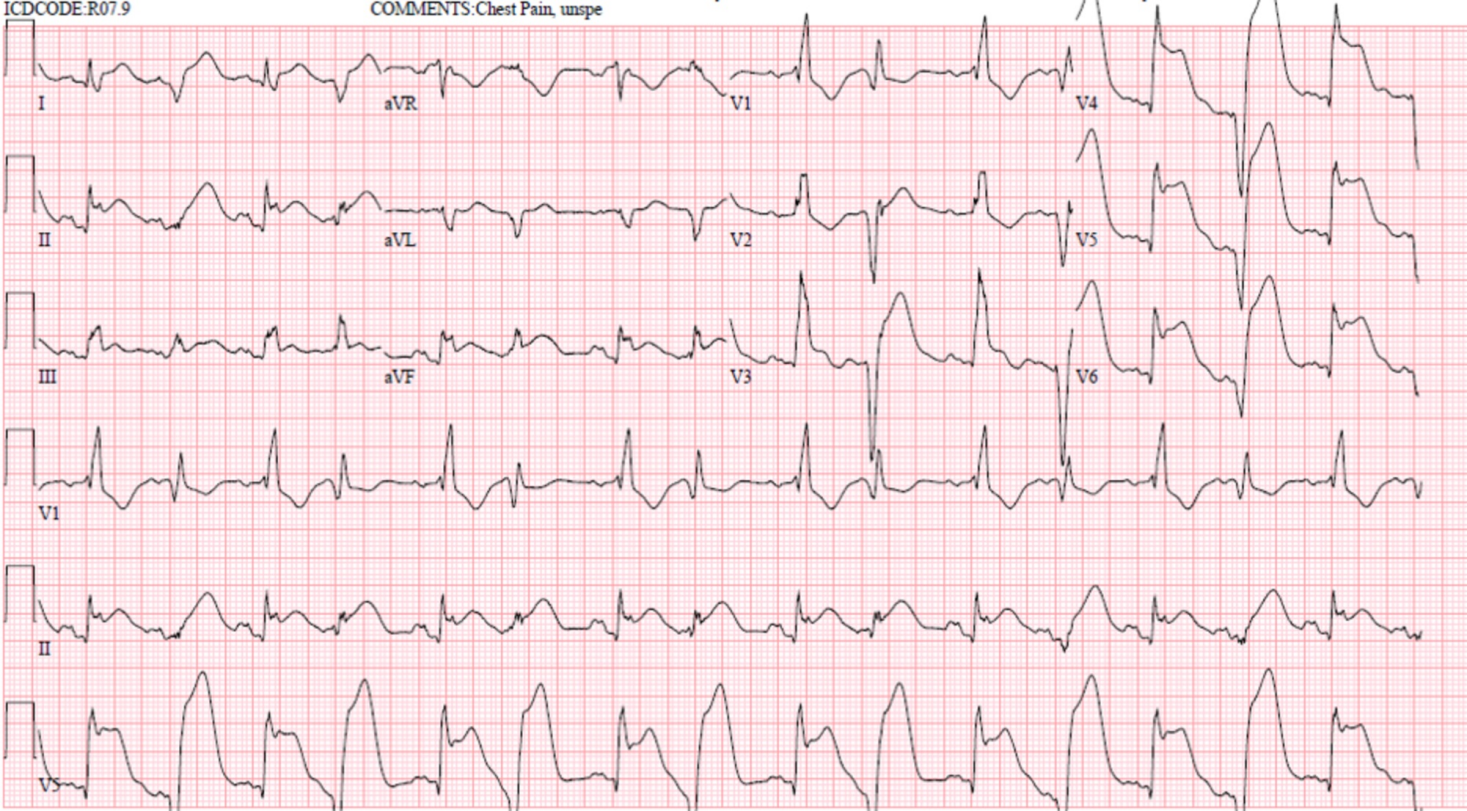
Electrocardiogram (EKG) of the patient, done after the patient complained of chest pain, showing ST elevations

**Figure 2:**
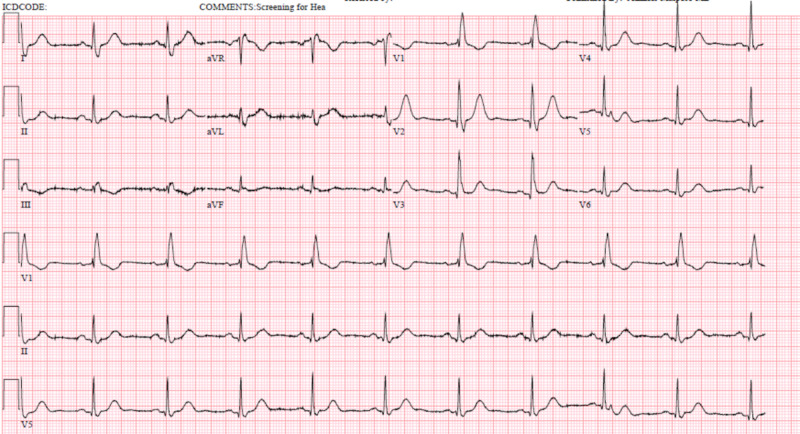
Baseline electrocardiogram (EKG) of the patient done when the patient initially presented to the emergency room

This is a case of an 88-year-old female with a pertinent medical history of lung cancer and paroxysmal atrial fibrillation. She initially came to us with the chief complaint of facial droop. She underwent extensive stroke workup that came out as negative for any acute findings. On further evaluation, she had an associated complaint of progressively worsening dysphagia for solid food over the last two weeks. This led to further workup and eventually she was diagnosed with new-onset paraneoplastic myasthenia gravis after her serologic testing came out positive. She was started on prednisone 40 mg and pyridostigmine 60 mg every six hours. She had a remarkable improvement in her dysphagia, unfortunately, a day later, she developed chest pain suddenly. It was severe in intensity, pressure like in nature, and radiating towards her left arm. The 12-lead electrocardiogram (EKG) done for chest pain, as seen in Figure 1, showed diffuse ST-elevations which had significantly changed from her previous baseline EKG done on initial presentation to the emergency department (Figure 2). We checked for her cardiac enzymes and they were found to be elevated as well [2,3]. Pyridostigmine was hence stopped, and a drug-eluting stent was placed in the left anterior descending artery (LAD) which showed 70% stenosis after the patient underwent cardiac catheterization. The patient was monitored in the cardiac care unit (CCU) for 24 hours, downgraded to the Medicine floor, and later on discharged home on oral prednisone.

## Discussion

Myasthenia gravis is an autoimmune disorder mainly caused by antibodies to the muscle acetylcholine receptors at the neuromuscular junction. Loss of these receptors leads to a defect in neuromuscular transmission with muscle weakness and fatigue.

Cardiovascular adverse events in myasthenia gravis and its treatment are extremely rare. To the best of our knowledge, only four cases of coronary vasospasm as a side effect of anticholinesterase medication have been reported [4,5].

We present a case of myocardial infarction in a patient with myasthenia gravis. Its prevalence varies between 1% to 12% only. The treatment for myasthenia gravis includes anti-cholinesterase therapy as a first-line therapy followed by steroids and immunomodulatory therapies. Our patient developed myocardial infarction with persistent ST wave elevation soon after being started on pyridostigmine. The exact mechanism under which vasospasm during treatment with acetylcholinesterase inhibitors occurs remains unknown. However, it is well recognized that the coronary artery response to acetylcholine is very sensitive and results in vasoconstriction and even vasospasm in some cases. Coronary vasospasm or angina can be provoked by methacholine or early morning exercise. This effect is not seen in patients pretreated with atropine sulfate [6-8]. In normal healthy individuals, acetylcholine is known to cause the release of nitric oxide from the endothelial cells leading to vasodilation [9,10]. However, when endothelial cells are lost or damaged, the reaction to acetylcholine is reversed leading to a contraction of coronary vessels, since nitric oxide cannot be produced anymore. Also in the presence of atherosclerotic lesions and hypercholesterolemia, the vasodilatory endothelial response to acetylcholine is diminished [11,12].

It is important for all the clinicians to be familiar with complications associated with anticholinesterase medications before starting patients on the treatment. Extra care should be taken in the case of elderly patients with underlying atherosclerosis, hypercholesterolemia, and hypertension [12]. Timely diagnosis and intervention play a pivotal role, shall any of these complications take place.

## Conclusions

This case indicates that even though anti-cholinesterase therapy is used as first-line therapy in patients with myasthenia gravis, we should be extremely cautious with their use, especially in the elderly population. It has been well recognized in the literature that the coronary arteries are very sensitive to acetylcholine. Coronary vasospasm is magnified in patients with underlying coronary artery disease; therefore, anti-cholinesterase drugs should be used very carefully in these patients. Anti-cholinesterase therapy, ideally if started in elderly patients and those with underlying coronary artery disease, should be in a controlled setting. Lastly, a strong clinical suspicion in regards to myocardial infarction should be there if the patient started on anti-cholinesterase therapy recently is complaining of chest pain. Myocardial infarction, if detected and treated early, can help us save the patient's life.
